# Epidemiological and genomic characterization of community-acquired *Clostridium difficile* infections

**DOI:** 10.1186/s12879-018-3337-9

**Published:** 2018-08-31

**Authors:** Christina S. Thornton, Joseph E. Rubin, Alexander L. Greninger, Gisele Peirano, Charles Y. Chiu, Dylan R. Pillai

**Affiliations:** 1 Department of Microbiology and Infectious Diseases, University of Calgary, Calgary, AB Canada; 2 Department of Medicine, University of Calgary, Calgary, AB Canada; 3 Calgary Laboratory Services, Calgary, AB Canada; 4 Department of Veterinary Microbiology, University of Saskatchewan, Regina, Canada; 5 Department of Laboratory Medicine, University of California San Francisco, San Francisco, California USA; 6 Department of Pathology and Laboratory Medicine, University of Calgary, Calgary, AB Canada; 7 Department of Laboratory Medicine, University of Washington, Seattle, WA USA; 8 Department of Medicine, Division of Infectious Diseases, University of California San Francisco, San Francisco, CA USA; 9 Diagnostic and Scientific Center, Room 1W-416, 9-3535 Research Road NW, Calgary, AB T2L 2K8 Canada

## Abstract

**Background:**

*Clostridium difficile* infection (CDI) is a major cause of morbidity and mortality in North America and Europe. The aim of this study was to identify epidemiologically-confirmed cases of community-acquired (CA)-CDI in a large North American urban center and analyze isolates using multiple genetic and phenotypic methods.

**Methods:**

Seventy-eight patients testing positive for *C. difficile* from outpatient clinics were further investigated by telephone questionnaire. CA-CDI isolates were characterized by antibiotic susceptibility, pulsed-field gel electrophoresis and whole genome sequencing. CA-CDI was defined as testing positive greater than 12 weeks following discharge or no previous hospital admission in conjunction with positive toxin stool testing.

**Results:**

51.3% (40/78) of the patients in this study were found to have bona fide CA-CDI. The majority of patients were female (71.8% vs. 28.2%) with 50–59 years of age being most common (21.8%). Common co-morbidities included ulcerative colitis (1/40; 2.5%), Crohn’s disease (3/40; 7.5%), celiac disease (2/40; 5.0%) and irritable bowel syndrome (8/40; 20.0%). However, of 40 patients with CA-CDI, 9 (29.0%) had been hospitalized between 3 and 6 months prior and 31 (77.5%) between 6 and 12 months prior. The hypervirulent North American Pulostype (NAP) 1-like (9/40; 22.5%) strain was the most commonly identified pulsotype. Whole genome sequencing of CA-CDI isolates confirmed that NAP 1-like pulsotypes are commonplace in CA-CDI. From a therapeutic perspective, there was universal susceptibility to metronidazole and vancomycin.

**Conclusions:**

All CA-CDI cases had some history of hospitalization if the definition were modified to health care facility exposure in the last 12 months and is supported by the genomic analysis. This raises the possibility that even CA-CDI may have nosocomial origins.

## Background

*Clostridium difficile* infections (CDI) are the most common cause of infectious diarrheal infection amongst hospitalized patients in North America and Europe [1]. CDI has recently surpassed methicillin-resistant *Staphylococcus aureus* as a hospital acquired infection [2, 3]. The incidence, mortality and associated health care costs associated with CDI are significant, with 10–25% of all cases of antibiotic-associated diarrheal onset attributed to CDI [4]. Clinically, nosocomial CDI has been well-studied with several risk factors for acquisition including hospitalization at time of infection, prior hospitalization, older age (> 65), antibiotic therapy (in particular fluoroquinolones, cephalosporins and clindamycin [5, 6]), use of nasogastric tubes, surgical procedures in the gastrointestinal tract, history of inflammatory bowel disease (IBD) and other states of immunosuppression [6, 7]. The pathogenicity of CDI is primarily attributed to the production of two exotoxins, toxin A and B that cause subsequent clinical manifestation including colonic dysregulation and cellular death [8]. A “binary” toxin has also been reported that may further contribute to pathogenesis in certain strains [1]. Additionally, CDI is spore-forming, allowing survival in the physical environment for prolonged periods of time [9], which challenges infection control and prevention in the hospital setting. Several other factors including gut microbiota disruption by antibiotic use and host endogenous antibodies to the toxins have also been implicated in CDI disease progression [6, 7, 10].

Recently, several studies have described the onset of community-acquired CDI (CA-CDI) [6]. The definition of CA-CDI requires the patient to not have been in a hospital or health care facility within the previous 12 weeks or to develop CDI symptoms within 48 h of hospital admission [11]. In contrast, nosocomial CDI requires that symptoms occur greater than 48 h after hospital admission or in less than 4 weeks after discharge from a health care facility [11]. Finally, indeterminate cases occur in the community between 4 and 12 weeks after hospital discharge has occurred [11]. CA-CDI rates are on the rise, with 20–45% [1, 9] of all CDI cases attributed to community onset, and a further 22% of patients having no history of antimicrobial several months prior to CDI onset [12].

CA-CDI rates amongst the pediatric population have also increased over time [13]. The clinical significance of *C. difficile* detection in infants and young children less than two years old is unclear, as they have been established as asymptomatic carriers [14]. Nevertheless, children may still serve as a means of transmission to other individuals in the household by exposure to spores within the same physical environment [15]. Exposure to *C. difficile* in the outpatient setting may serve as a means of transmission [16]. Two-thirds of CA-CDI patients had some form of occupational exposure within health care fields that may have preceded their illness [17]. There is also evidence that food-borne exposure to *C. difficile* may be a means of transmission within the community as spores have been demonstrated to survive normal cooking temperatures [18]. Zoonotic reservoirs exist in several animals including cattle and pigs [15, 19–23].

The aim of this study was to better understand the impact of CA-CDI by identifying risk factors linked to epidemiologically confirmed CA-CDI cases in the metropolitan center of Calgary (approximately 1.3 million people). *C. difficile* isolates from CA-CDI cases were further characterized by antibiotic susceptibility testing, pulsed field gel electrophoresis and whole genome sequencing.

## Materials and methods

### Study population

Patients whose stool specimens were submitted from patient service centers to Calgary Laboratory Services and positive for *C. difficile* toxin between March and October 2012 were included in the study. These patients were included based on physician suspicion of *C. difficile* infection and test submission. The physician decision to test was not scrutinized by the study team but taken at face value. A control group of nosocomial strains were collected from inpatients admitted during the study period. All stools were diarrheal. A nosocomial strain was defined as a stool specimen positive for *C. difficile* from a patient admitted to one of the four major hospitals in the city of Calgary. Medical records including hospitalizations, relevant laboratory tests and diagnosis was obtained through a centralized provincial database. Ethical approval was obtained through University of Calgary Conjoint Health Research Ethics Board (ID 13–0406).

### Telephone survey

For the purposes of this study, CA-CDI was defined as infection occurring greater than 12 weeks following discharge or no previous hospital admission. CA-CDI patients (*n* = 78) were, within 12 months of testing positive, assessed by telephone questionnaire for epidemiological data related to the mode of acquisition. The telephone study was designed to gauge risk factors traditionally associated with CDI acquisition reported in the literature. For patients who could not recall their last hospitalization, an electronic medical record (EMR) was interrogated to determine the date and site of visit if any.

### Specimen collection, testing, culture, and deoxyribonucleic acid (DNA) isolation

Patients’ stools were tested for CDI using a two-step testing algorithm which includes GDH screening by ELISA (Diasorin LIAISON® *C. difficile* GDH) followed by toxin testing (Cepheid Xpert® *C. difficile*/Epi) from outpatient physician offices. *C. difficile* isolates were selected on cycloserine cefoxitin fructose agar (CCFA) and species confirmed by matrix assisted laser desorption ionization-time of flight mass spectrometry (MALDI-TOF MS) Genomic DNA was extracted for whole genome sequencing using an in-house modified protocol from the QIAamp DNA Mini Extraction Kit (Qiagen, USA). Colonies were re-suspended in 0.9% saline to a McFarland standard of 2.4–3.0 and subsequently centrifuged at 13,000 rpm (rpm) on a tabletop centrifuge for one minute with supernatant discarded. The remaining pellet was re-suspended in 180 μL of enzyme solution (20 mg/mL lysozyme, 20 mM Tris-HCl, 2 mM EDTA and 1.2% Triton X). The solution was then incubated for 30 min at 37 °C until clearing of the solution was noted. Twenty μL of reconstituted Proteinase K solution (Sigma-Aldrich, USA) was added with a further incubation at 56 °C overnight. The remainder of the protocol followed instructions as denoted in the manufacturer’s protocol. The final pellet was re-suspended in 50 μl DNase/RNase free water.

### Antibiotic susceptibility testing

In brief, cultured isolates grown on agar plates for 48 h were suspended in 0.9% saline and standardized to a 1.0 McFarland density. The inoculum was then evenly spread over blood agar and incubated in anaerobic incubation conditions for 24 h. Metronidazole and vancomycin E-test strips (BioMerieux, USA) were stored at -20 °C until use. Following inoculation of plates, E-test strips were then placed and incubated for a further 48 h before measurement. Epidemiological cut-off (ECOFF) guidelines were used from EUCAST with the following breakpoint: vancomycin > 2 mg/L as non-susceptible and < 2 mg/L as susceptible; metronidazole > 2 mg/L as non-susceptible and < 2 mg/L as susceptible [24]. E-test strips were used to correlate with disk diffusion based on prior research demonstrating adequate agreement [25].

### Pulsed-field gel electrophoresis (PFGE)

PFGE was conducted on CA-CDI isolates as outlined in previous methods [26, 27]. Briefly, DNA was prepared by lysis of cells within agarose plugs. The resultant plugs were then digested with *Sma*I as described previously [28]. A BioRad Chef Mapper was used to visualize the resulting fragments [27]. PFGE profiles were then compared using BioNumerics, version 5.1 (Applied Maths, Belgium) with standardized *C. difficile* NAP serotypes that were run as controls in parallel with samples. The resulting dendograms were then categorized using an unweighted-pair group method using average linkages (UPGMA), Dice similarity coefficient (optimization 1.5%, tolerance 1%) [28, 29]. Clustering of PFGE results was performed using Advanced Cluster Analysis algorithm from BioNumerics, version 5.1 (Applied Maths, Belgium).

### Whole genome sequencing

Next-generation sequencing libraries were prepared using quarter-volume reactions of Nextera XT (Illumina) and 1 nanogram of genomic DNA with 14 cycles of amplification. Barcoded libraries were pooled and sequenced on an Illumina HiSeq 2x250bp (nosocomial isolates; control group) and the Illumina MiSeq (CA-CDI isolates; study group). Two different sequencers were used due to logistical reasons. Only a subset of CA-CDI, indeterminate, and nosocomial isolates (*n* = 71) were sequenced due to study budgetary constraints. Paired-end reads were run through the nullarbor (https://github.com/tseemann/nullarbor) pipeline using the *C. difficile* 630 genome (NC_009089) as a reference. Phylogenetic trees were constructed based on the core genome SNP alignment using MrBayes (http://mrbayes.sourceforge.net/). The dendogram was viewed and exported using FigTree (http://tree.bio.ed.ac.uk/software/figtree/).

## Results

Patients (*n* = 78) testing positive for CDI from outpatient physician offices were contacted by telephone questionnaire to assess epidemiological risk and exposures in detail. Further characterization based on clinical timelines reclassified patients into CA-CDI, defined as greater than 12 weeks following discharge or no previous hospital admission (40/78; 51.2%; Table 1). Overall, the majority of patients were female (71.8% vs. 28.2%) and between 50 and 59 years of age (21.8%).

**Table 1:** Demographics of Patients Contacted for Community *C. difficile* Study

Demographic Characteristics	CA-CDI Strains^a^
Total	40 (51.2)
Gender	
Male	11 (27.5)
Female	29 (72.5)
Age	
< 10	1 (2.5)
10–19	1 (2.5)
20–29	6 (15.0)
30–39	2 (5.0)
40–49	1 (2.5)
50–59	8 (20.0)
60–69	10 (25.0)
70–79	5 (12.5)
> 80	6 (15.0)

^a^Defined as > 12 weeks following discharge or no previous hospital admission. CA-CDI; community-acquired *C. difficile* infection

The most common co-morbidities in CA-CDI patients included ulcerative colitis (1/40; 2.5%), Crohn’s Disease (3/40; 7.5%), celiac disease (2/40; 5.0%) and irritable bowel syndrome (8/40; 20.0%) (Table 2). Among the 40 patients with CA-CDI, 31 (77.5%) had no recent hospitalizations within the last 6 months prior to infection. Twenty-one patients (52.5%) had a household contact admitted to the hospital the year prior to onset of CDI. Seventeen patients (42.5%) had a household contact that had confirmed CDI in the year prior.

**Table 2:** Telephone Survey Results of Patients Contacted for Community *C. difficile* Study

	CA-CDI (%)^a^
Total	40 (51.2)
Prior diagnosis of CDI	15 (37.5)
Most recent hospitalization:	
> 6 months	31 (77.5)
Within 6 months	9 (22.5)
Within 3 months	0 (0.0)
Previous week	0 (0.0)
Times hospitalized in last 6 months:	
1	0 (0.0)
2–5	7 (17.5)
5–10	2 (5.0)
Prior history of Ulcerative Colitis	1 (2.5)
Prior history of Crohn’s Disease	3 (7.5)
Prior history of Celiac disease	2 (5.0)
Prior history of IBS	8 (20.0)
Institutional care in prior year	5 (12.5)
Visit someone in nursing home or hospital prior year before CDI	17 (42.5)
Household contact in hospital in prior year before CDI infection	21 (52.5)
Occupation in health care field	4 (44.4)
Work with patients diagnosed with CDI in prior year	1 (2.5)
Household contact hospitalized in year prior to CDI infection	17 (42.5)
Household contact with CDI infection in year prior to CDI	9 (22.5)
Children in household	
No	15 (37.5)
0–1 year	8 (20.0)
1–2 years	3 (7.5)
2–5 years	10 (25.0)
7–10 years	20 (50.0)
Household pets:	
No	16 (40.0)
Dogs	9 (22.5)
Cats	4 (10.0)
Fish	2 (5.0)
Birds	5 (12.5)
Other	5 (12.5)
Occupational work related to pork products	5 (12.5)
Consume pork products	
No	12 (30.0)
Yes, 1 time per week	7 (17.5)
Yes, 2–4 times per week	11 (27.5)
Yes, > 4 times per week	10 (25.0)
Vegetarian	8 (20.0)

^a^Defined as > 12 weeks following discharge or no previous hospital admission. CA-CDI; community-acquired *C. difficile* infection

**Fig. 1:**
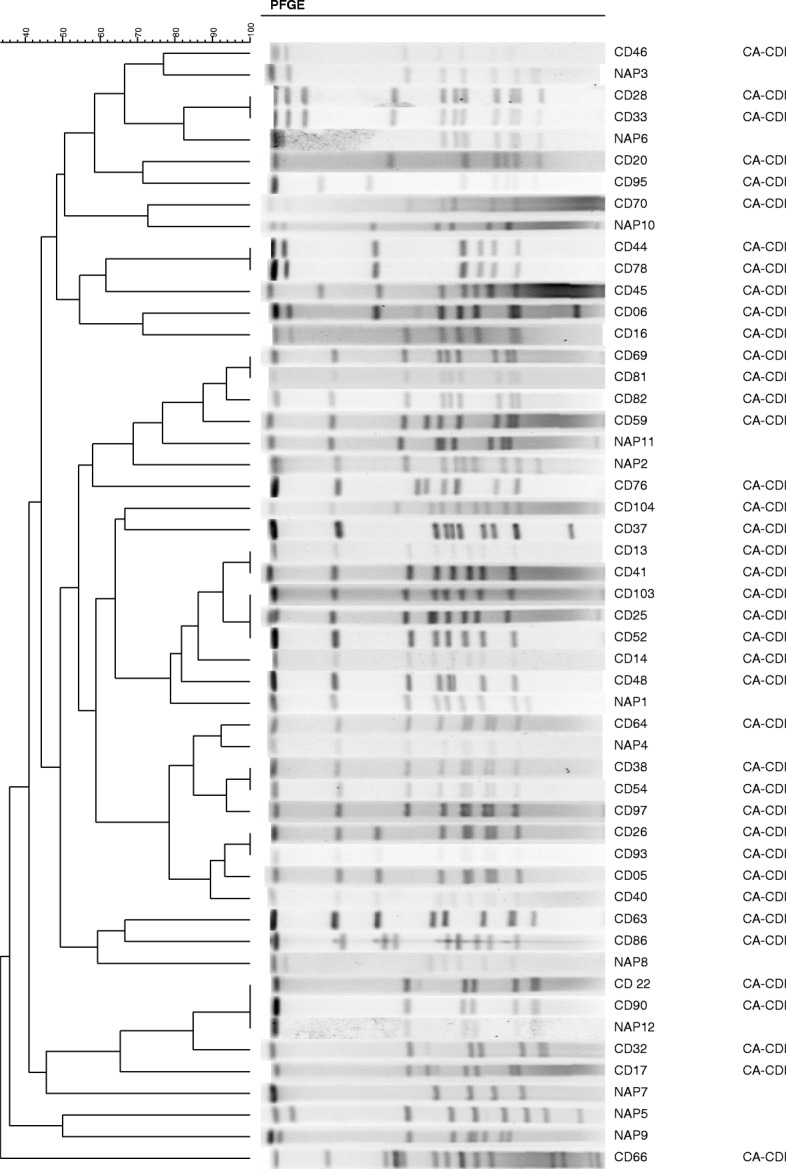
Pulsed field gel electrophoresis from epidemiologically confirmed community- acquired *C. difficile* isolates (CA-CDI labelled “CD”) compared to reference North American pulsotypes (labelled “NAP”)

PFGE analysis was done on CA-CDI isolates collected during the study. Figure 1 highlights the CA-CDI PFGE pulsotypes. Amongst the CA-CDI isolates, both NAP1 (9/40; 22.5%) and NAP 4 (8/40; 20%) were the most abundant, followed by NAP 2/11 (5/40; 12.5%), NAP 12 (4/40; 10%), and NAP 6 (4/40; 10.0%) (Table 3). Antibiotic susceptibility for metronidazole and vancomycin was tested for CA-CDI isolates received from the community. There was universal susceptibility amongst all isolates to metronidazole and vancomycin based on accepted interpretive breakpoints (data not shown).

**Table 3:** Community acquired (CA) *C. difficile* Isolates (*n* = 40) categorized by related North American Pulsotype (NAP)

NAP Type	CA-CDI (%)^a^
NAP1	9 (22.5)
NAP2/11	5 (12.5)
NAP3	1 (2.5)
NAP4	8 (20.0)
NAP5	0 (0.0)
NAP6	4 (10.0)
NAP7	0 (0.0)
NAP8	0 (0.0)
NAP9	0 (0.0)
NAP10	0 (0.0)
NAP12	4 10.0)
Non-NAP	9 (22.5)

^a^Defined as greater than 12 weeks following discharge

**Fig. 2:**
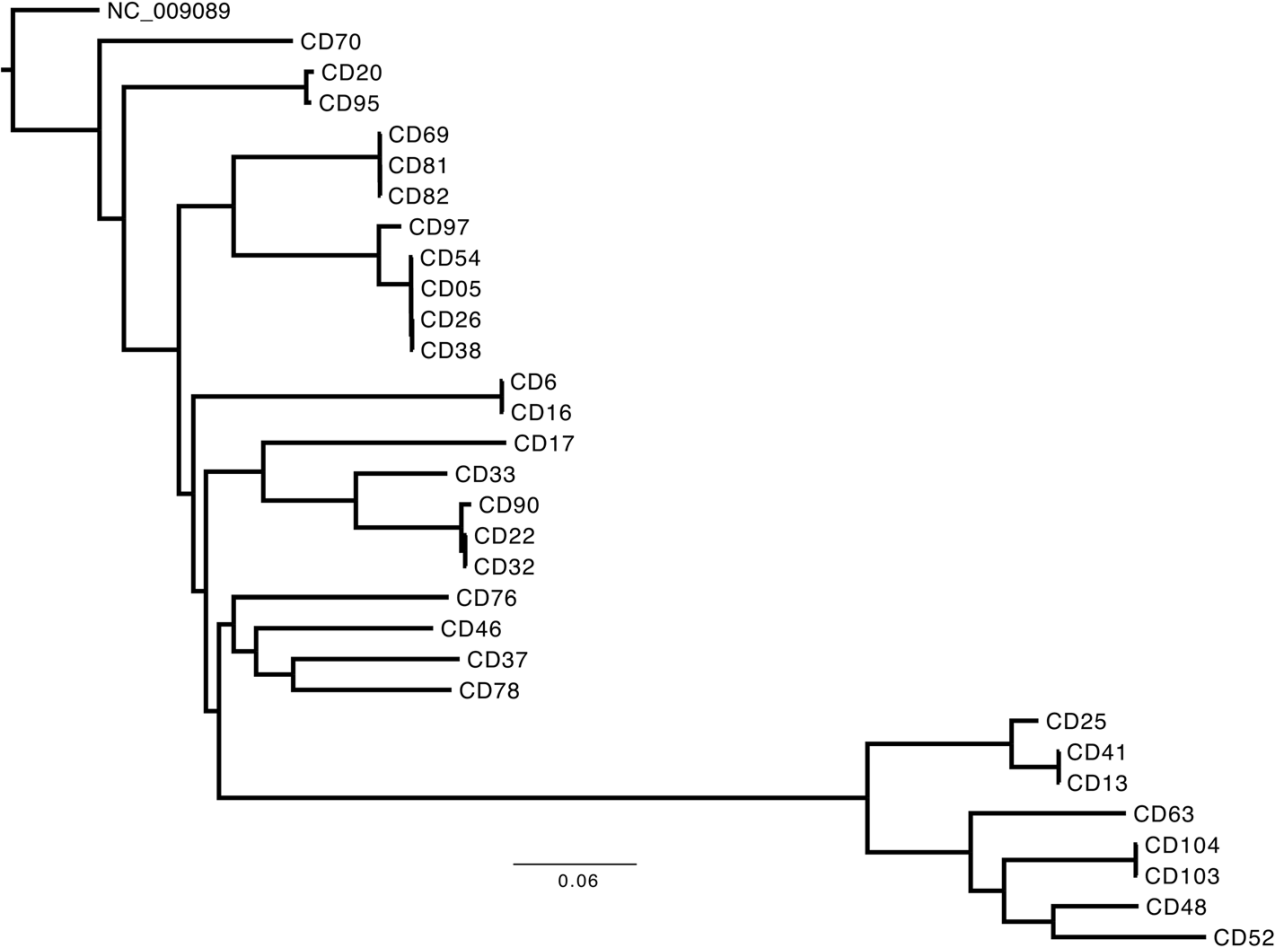
Core genome SNP phylogeny of community-acquired isolates (“CD”) sequenced in this study. Reads from 30 community-acquired *C. difficile* isolates (CA-CDI) were run through the nullarbor pipeline using *C. difficile* 630 genome (NC_009089) as the reference and core SNP genome phylogenies were constructed using MrBayes using the 630 reference genome as the outgroup. A NAP-1 like cluster (CD25..CD52) is noted. The bootstrap support values for all branches are 100

Whole genome sequencing (WGS) was performed on a subset of epidemiologically confirmed CA-CDI isolates (*n* = 30). Figure 2 demonstrates the phylogenetic tree derived from whole genome sequencing of CA-CDI isolates. Concordance between PFGE and WGS was observed based on phylogenetic analysis in terms of clustering with reference North American pulsotypes. A NAP-1 like cluster (CD25, CD41, CD13, CD63, CD104, CD103, CD48 and CD52) was noted from the WGS phylogenetic tree (bottom of Fig. 2). This cluster isolates represented patients with no hospitalizations in the six months prior to positive stool toxin, suggesting the possibility of a community-acquired NAP-1 like cluster. There was no other significant epidemiological association noted between these patients.

**Fig. 3:**
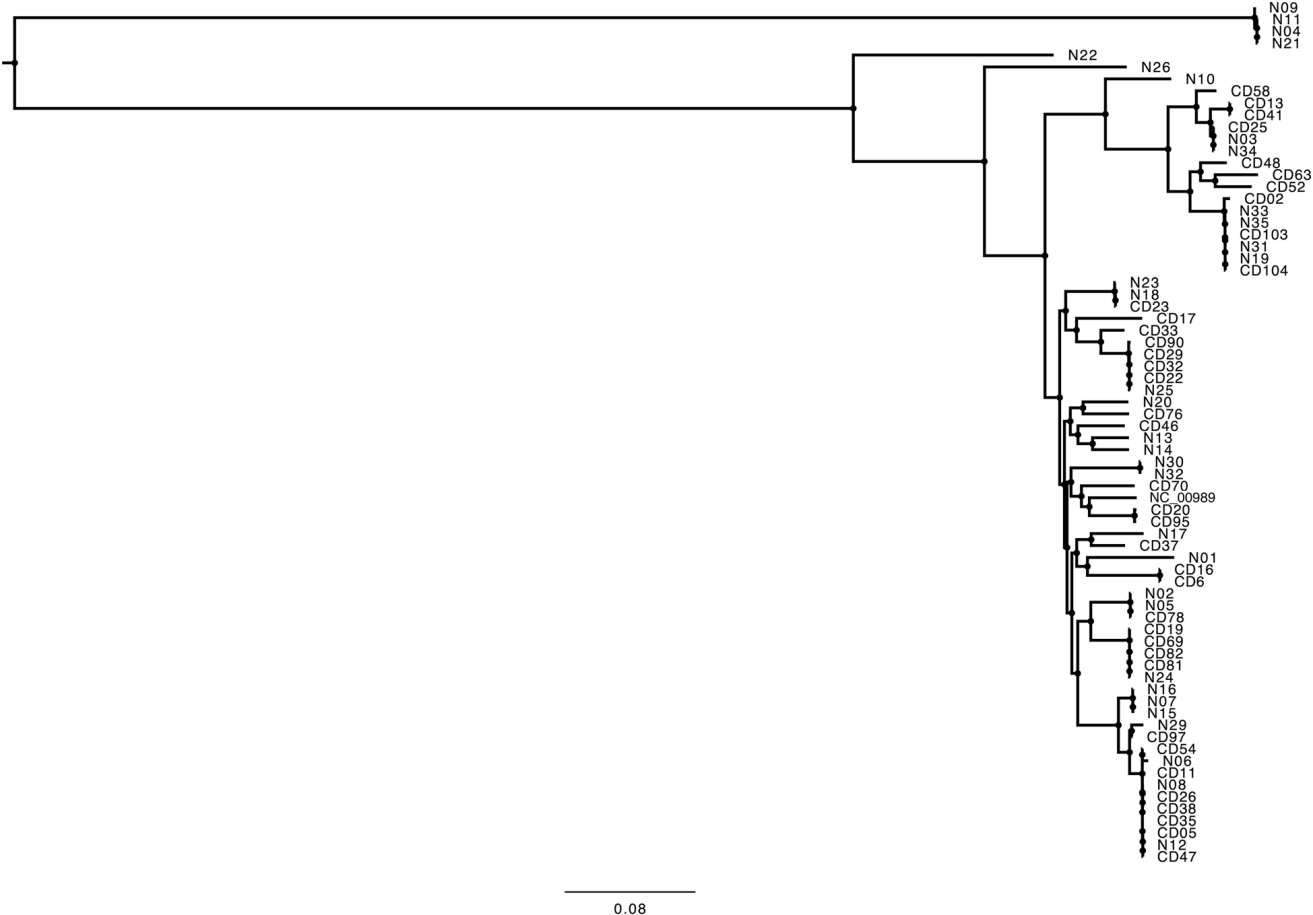
Core genome SNP phylogeny from community-acquired (*n* = 30) and indeterminate (*n* = 8) (“CD”), and nosocomial (“N”) *C. difficile* isolates (*n* = 33) was constructed as described in Fig. 2 using *C. difficile* 630 genome (NC_009089) as the reference. This tree was midpoint rooted based on the long genetic distance present between the majority of isolates and four nosocomial isolates (N09, N11, N04, N21). The bootstrap support values for all branches are 100

In order to determine whether unique clusters exist in CA-CDI (*n* = 30) as compared to indeterminate isolates (*n* = 8) from this study or a set of nosocomial isolates (*n* = 33) collected from the hospitals during the study period, a WGS phylogenetic analysis was performed. Nosocomial isolates were obtained from a separate control group of patients admitted to hospital during the study period. A phylogenetic tree comparing all three epidemiological categories is presented in Fig. 3, demonstrating that CA-CDI and nosocomial strains are genomically indistinguishable.

## Discussion

CA-CDI is associated with significant clinical complications. It is estimated that 40% of patients require hospitalization, 20% suffer severe infection, 4.4% go on to have severe complications, 20% end up with treatment failure, and 28% have recurrent CDI [30]. An economic analysis from Canada found nearly 38,000 cases of CDI in 2012 with a total cost to society of $281 million, in part due to in-hospital investigations, community management and productivity loss [31]. Furthermore, management of CDI relapses alone was over $65 million. This has tremendous impact on the health care system in terms of costs, patient outcomes and burden on already limited resources [15]. We undertook to closely examine CA-CDI using a patient-based telephone questionnaire, to better understand this epidemiological group. We also analyzed the isolates using multiple laboratory methods to determine if unique traits associated with CA-CDI such as antibiotic resistance or genetic features exist.

Several studies have assessed epidemiology and risk factors associated with CA-CDI. One recent study from Japan [32] found the average age to be 58.8 with 50% > 65 years of age, similar to our findings. The gender distribution was also noted to be similar between studies (26.9% male). Interestingly, this study found the majority of patients were more likely to have received antimicrobials (oral fluoroquinolones most notably) and antacids. A recent report from the US found 40% of patients with CA-CDI were not previously exposed to antimicrobials [33]. Our study did assess antimicrobial prescription through the telephone survey but resulted in a large proportion of patients unable to recall the timeframe of antibiotic use. The most striking epidemiological finding in the current study is that all CA-CDI cases had some history of hospitalization if the definition were modified to hospital exposure in the last 12 months rather than the last 12 weeks as currently accepted. This raises the possibility that even CA-CDI has nosocomial origins albeit remote.

Metronidazole and vancomycin are considered first line agents in the clinical treatment of nosocomial *C. difficile* infection. The susceptibility observed with both antibiotics in our study suggests antibiotic resistance in CA-CDI is not a predisposing factor. There have been some reports [34] and in vitro data [35] suggestive of antibiotic resistance in nosocomial CDI, but limited data exists for CA-CDI. Furthermore, increasing resistance has been reported in toxigenic *C. difficile* strains. A study from Spain found resistance to metronidazole and intermediate resistance to vancomycin amongst nosocomial CDI, albeit with none resulting in treatment failure [36]. Ultimately, as antibiotic exposure increases in the CA-CDI population, it will be necessary to further monitor resistance patterns and assess clinical utility of standard antibiotic treatment regimens.

Molecular characterization by PFGE analysis and WGS demonstrated the predominance of the NAP1 ribotype in CA-CDI. The NAP1 hypervirulent strain has been associated with increased toxin production within animal models and increased antibiotic resistance, in particular to fluoroquinolones [37]. Reports exist of increased detrimental clinical outcomes including intensive care admission, requirement for colectomy, or death with this strain [38, 39]. Clinical reports of the hypervirulent NAP1 strain have been primarily associated thus far with nosocomial *C. difficile* infection. The presence of NAP 1 in the community with no recent hospital exposure implies that this strain can enter the nosocomial setting upon admission. Our WGS analysis suggests that CA-CDI isolates are indistinguishable from nosocomial isolates and no unique cluster is especially adapted to the community. CA-CDI essentially mirrors nosocomial isolates at the genomic level in this study. Microbiome analysis has been well elucidated in nosocomial infections, with significant dysbiosis shown in patients following infection [40–42]. Dysbiosis may also be a predisposing factor in the acquisition of CDI not only in the hospital but also in the community.

Limitations of this study include the small sample size and number of patients lost to follow-up when contacted by telephone survey. As well, community clinics are within the vicinity of our hospitals and there is a possibility that these patients had some kind of incidental hospital contact in recent weeks (i.e. less than 12). A study performed on CA-CDI in a rural community where no hospitals are in the vicinity would rule this possibility out. Further limitations include data only acquired from a single centre (at least geographically); limited patient-level data available; observational study design; lack of inclusion of hospitalized patients with CA-CDI; lack of ability to distinguish CDI from *C. difficile* colonization; and recall or memory with a timespan of months between the occurrence of CDI and the telephone interview. Longitudinal studies may be of value where patients in the community are tested and followed over time in a prospective fashion. Nevertheless, there is a paucity of epidemiological and strain typing data on CA-CDI and these results support a community-nosocomial linkage even in cases traditionally attributed to the community.

## Abbreviations

CA: Community acquired
CDI: *Clostridium difficile* infection
DNA: Deoxyribonucleic acid
ECOFF: Epidemiological cut-off
IBD: Inflammatory bowel disease
PFGE: Pulsed field gel electrophoresis
WGS: Whole genome sequencing
